# Complications traumatiques et psychosociales des chutes chez le sujet âgé tunisien

**DOI:** 10.11604/pamj.2019.32.92.16667

**Published:** 2019-02-26

**Authors:** Ines Kechaou, Eya Cherif, Ben Salem Sana, Imène Boukhris, Lamia Ben Hassine

**Affiliations:** 1 Service de Médecine Interne B, Hôpital Charles Nicolle, Université de Tunis El Manar, Faculté de Médecine de Tunis, Tunisie

**Keywords:** Sujet âgé, chute, conséquences, fracture, Elderly, fall, consequences, fracture

## Abstract

**Introduction:**

Les chutes chez le sujet âgé constituent un problème de santé majeur de par les complications traumatiques et psychosociales qui peuvent entrainer une perte de l'autonomie et un état de dépendance. L'objectif de notre travail est d'étudier les circonstances et les conséquences traumatiques et psychosociales des chutes chez les sujets âgés.

**Méthodes:**

Nous avons mené une étude rétrospective réalisée entre septembre 2014 et janvier 2016 incluant 40 patients âgés de 65 ans et plus ayant fait au moins une chute l'année précédente recrutés parmi les patients hospitalisés ou suivis à la consultation externe du service de Médecine Interne B de l'hôpital Charles Nicolle. Les circonstances et les conséquences des chutes ont été recueillies par l'interrogatoire rétrospectif des patients et de leur entourage à distance de la chute.

**Résultats:**

L'âge moyen des patients chuteurs était de 75,7 ans avec une prédominance féminine nette (30F/10H). Des facteurs précipitants étaient retrouvés chez 38 patients. Ils étaient de type extrinsèque dans 78,9% des cas et intrinsèques dans 50% des cas. Un séjour prolongé au sol était noté dans 10% des cas. Les fractures étaient plus fréquentes chez les femmes (12F/1H) intéressant surtout les membres supérieurs (61,5%). Les conséquences psychosociales ont été plus fréquentes chez les femmes. Un syndrome post chutes a été noté chez 5 patients.

**Conclusion:**

La correction des facteurs précipitants intrinsèques et extrinsèques des chutes et l'apprentissage du relever du sol en post chutes permettra de prévenir le risque de chute ainsi que ses conséquences graves.

## Abstract (en)

**Introduction:**

Falls in the elderly are a major health problem due to their traumatic and psychosocial complications which may lead to a loss of autonomy and a state of dependency. This study aims to study the traumatic circumstances and psychosocial consequences of falls in the elderly.

**Methods:**

We conducted a retrospective study of 40 patients aged 65 years and over with a history of at least one fall in the previous year between September 2014 and January 2016. They were enrolled among patients hospitalized or treated on an outpatient basis in the Department of Internal Medicine B in the Hospital Charles Nicolle. Data on circumstances and consequences of falls were collected through a retrospective interview with patients and their entourage about the fall.

**Results:**

The average age of fallen patients was 75,7 years, with a female predominance (30F/10M). Precipitating factors were found in 38 patients. Extrinsic factors were found in 78.9% of cases while intrinsic factors in 50% of cases. Extended stay on the ground was reported in 10% of cases. Fractures were more frequent among women (12F/1M), especially involving upper limbs (61.5%). Psychosocial consequences were more frequent among women. Post-fall syndrome was found in 5 patients.

**Conclusion:**

The correction of the extrinsic and intrinsic precipitating factors of falls and the implementation of educational programs on how elderly should get up from the ground properly after a fall will prevent the risk of a fall as well as fall-related severe consequences.

## Introduction

Les chutes chez le sujet âgé représentent un problème mondial et probablement national [1-3]. Elles représentent la 5^ème^ cause commune de décès en gériatrie et sont considérées parmi les causes classiques de décès chez les sujets âgés de plus de 75 ans [4, 5]. En effet, après une chute, le décès survient dans 11% des cas [6]. Le risque de décès est plus important quand le temps passé au sol est élevé. En effet, 50% des chuteurs ayant dépassé plus d'une heure au sol décèdent dans les 6 mois suivant la chute [7]. La morbidité secondaire aux chutes est liée d'une part au type de fractures et à la gravité des blessures. D'autre part, elle est liée aux conséquences psychosociales des chutes qui sont également importantes, mais peu étudiés vu la difficulté de les quantifier. Les admissions à l'hôpital pour chutes augmentent avec l'avancée en âge. En effet, elle est de 6 fois entre les sujets ayant un âge entre 65 et 69 ans et chez ceux de plus de 85 ans [4]. Nous présentons les résultats d'une analyse rétrospective d'un échantillon de patients tunisiens ayant un âge ≥65 ans dans laquelle notre objectif principal était d'étudier les circonstances des chutes ainsi que ses conséquences physiques et psychosociales. Notre objectif secondaire était de décrire le retentissement des chutes sur l'autonomie.

## Méthodes

Nous avons mené une étude rétrospective entre septembre 2014 et janvier 2016 incluant 40 patients âgés de 65 ans et plus ayant fait au moins une chute l'année précédente recrutés parmi les patients hospitalisés ou suivis à la consultation externe du service de médecine interne. Ont été inclus seulement les patients qui pouvaient nous procurer eux même ou les membres de leur famille les informations nécessaires concernant leurs chutes.

Notre étude a été basée sur l'interrogatoire rétrospectif des patients ainsi que de leur entourage visant à recueillir les informations concernant le nombre de chutes, les circonstances de la première chute, les facteurs précipitants extrinsèques (liés à l'environnement et à l'habitat) et intrinsèques (liés aux évènements aigus ayant entrainé un trouble de l'équilibre), le temps passé au sol, les conséquences physiques et psychosociales. En cas de consultation aux urgences après la chute, nous avons précisé les explorations réalisées. Le degré d'autonomie après la chute a été évalué grâce au score ADL (activity daily living). Le caractère répétitif des chutes a été considéré à partir du moment où la personne a fait au moins deux chutes sur une période de 12 mois [8].

### Analyse statistique

Les données ont été analysées au moyen du logiciel SPSS version 20. Dans notre étude, les fréquences simples ont été calculées pour les variables qualitatives. Les moyennes, les médianes et les écarts types ont été calculés pour les variables quantitatives.

## Résultats

Il s'agissait de 40 patients chuteurs ayant un âge moyen au moment de la chute de 75,7 ans (±5,8) avec des extrêmes de 65 à 88 ans. Un âge ≥70 ans a été retrouvé chez 36 patients (90%) et ≥80 ans dans 30% des cas. Nos patients étaient répartis entre 30 femmes et 10 hommes (sexe ratio: 0,33). Quatre patients vivaient seuls et 36 étaient au sein de leur famille. L'HTA (77,5%), l'arthrose (72,5%) et le diabète (65%) ont constitué les pathologies les plus fréquentes (Tableau 1). L'ostéoporose a été retrouvée chez 12 parmi 17 patients (70,6%) qui ont bénéficié d'une densitomètrie osseuse (DMO). Une polypathologie avec un nombre moyen de pathologies chroniques ≥3 était notée chez 24 patients (60%). Le nombre moyen des médicaments chez les chuteurs était de 4,4 (±2,6) avec des extrêmes allant de 1 à 9. Une polymédication ≥3 médicaments était notée chez 27 patients (67,5%). Un nombre de médicaments ≥4 médicaments était retrouvé chez 22 patients (55%). Il s'agissait surtout de médicaments à visée anti-hypertensive (77,5%), antidiabétique (65%) et hypolipémiants (42%). Le nombre moyen des chutes récentes dans l'année précédente était de 1,6 (±0,98) avec des extrêmes de 1 à 5. Des chutes répétées étaient notées chez 16 patients réparties entre 10 femmes et 6 hommes.

**Tableau 1:** Comorbidités chez les patients chuteurs

Types de pathologies	Nombre de patients/40 (%)
HTA	31 (77,5%)
Arthrose	29 (72,5%)
Diabète	26 (65%)
Dyslipidémie	17 (42,5%)
Ostéoporose	12/17 (70%)
Syndrome dépressif	7 (17,5%)
Hypothyroïdie	6 (15%)
Anémie ferriprive	6 (15%)
Asthme	4 (10%)
Insuffisance coronarienne	3 (7,5%)
BPCO	3 (7,5%)
AVC ischémique	2 (5%)
Insuffisance cardiaque	2 (5%)
Cancer du colon	1 (2,5%)
Fibrillation auriculaire	1 (2,5%)
Maladie de Biermer	1 (2,5%)
Myélome multiple	1 (2,5%)
Démence	1 (2,5%)
Maladie de Vaquez	1 (2,5%)
Sarcoïdose	1 (2,5%)
Polyangéite microscopique	1 (2,5%)
Goutte	1 (2,5%)
Dermatomyosite en rémission	1 (2,5%)
Canal lombaire étroit	1 (2,5%)

**BPCO**: bronchopneumopathie chronique obstructive, **AVC**: accident vasculaire cérébral

### Circonstances des chutes

Des facteurs précipitants étaient retrouvés chez 38 patients. Ils étaient de type extrinsèque chez 30 patients (78,9%) et intrinsèques chez 19 patients (50%) (Tableau 2). Parmi les causes extrinsèques, une origine environnementale a été identifiée chez 28 patients (73,6%) et vestimentaire chez 20 patients (52,6%). Pour les causes intrinsèques, l'hypotension orthostatique a été parmi les causes les plus fréquentes (Tableau 2). Une crise convulsive a été identifiée chez un seul patient âgé de 81 ans, en rapport avec des métastases cérébrales. Les malaises et les lipothymies étaient retrouvés chez 28 patients (70%) et étaient d'origine indéterminée dans 23,6% des cas (Tableau 2).

**Tableau 2:** Facteurs précipitants des chutes

Causes aiguës	Nombre de patients 38 (%)
Causes extrinsèques	**30 patients (78,9 %)**
Causes environnementales	**28 (73,6%)**
Escalier	13 (34,2%)
Revêtement glissant	4 (10,5%)
Obstacle	3 (7,8%)
Objet mal rangé	2 (5,2%)
Tapis	1 (2,6 %)
Fil électrique qui traine	1 (2,6 %)
Changement d’environnement	6 (15,7 %)
Causes vestimentaires	**20 (52,6%)**
Habits longs	15 (39,4%)
Chaussures inadaptées	5 (13,1%)
Causes intrinsèques	**19 (50%)**
Hypotension orthostatique	5 (13,1%)
Hypoglycémie	3 (7,8 %)
Crise convulsive	1 (2,6%)
Perte de connaissance prolongée (AVC ischémique)	1 (2,6%)
Lipothymie et /ou syncope d’origine indéterminée	9 (23,6%)

### Lieu de chutes

Les chutes sont survenues à domicile chez 36 patients (90%) et dans la rue chez 4 patients (10%). Parmi toutes les femmes, 27 ont chuté dans leur maison (90%) et parmi les hommes 1 seul a chuté à domicile (10%). La chute était en rapport avec un changement de l'environnement dans 6 cas (15%): maison de fille/voisine/cousine.

### Heure de chute

La plupart des chutes étaient survenues le jour (33 patients: 82,5%). Les 7 autres patients (17,5%) ont chuté la nuit.

### Séjour au sol et relever du sol

Après la chute, 15 patients soit 35,5% se sont relevés seuls. Le reste des patients soit 62,5% ont eu besoin d'aide d'une tierce personne pour se relever. Un séjour prolongé au sol supérieur à 1 heure a été rapporté par 4 patients (10%).

### Consultation et exploration des chutes

Parmi les patients chuteurs, 25 patients (62,5%) ont consulté aux urgences immédiatement après la chute. Parmi eux, 20 patients ont bénéficié d'un bilan radiologique standard, 11 d'un électrocardiogramme (ECG) et 6 d'un scanner cérébral en urgence. Quinze patients n'ont pas consulté dans les suites immédiates de la chute. Aucun des patients chuteurs n'a bénéficié d'une enquête étiologique pour une prise en charge spécifique des chutes.

### Conséquences traumatiques des chutes

Les fractures ont été les conséquences les plus fréquentes, retrouvées chez 13 patients (32,5 %). Elles ont été plus fréquentes chez les femmes (12F/1H) (Tableau 3). Le membre supérieur a été le site le plus fréquemment touché (8 patients). Une ostéoporose a été retrouvée chez 10 patients fracturés et qui ont été explorés par une densitométrie osseuse (DMO). Les détails des fractures sont illustrés dans le Tableau 3. Une complication à type de traumatisme crânien compliqué d'un hématome sous dural et d'une hémorragie méningée grave sans fracture a été notée chez 1 patient (2,5%) âgé de 78 ans en dehors de tout traitement anticoagulant. Des conséquences non graves ont été rapportées par 26 patients (65%). Il s'agissait d'hématomes sous cutanés dans 19 cas (47,5%), et de plaies superficielles dans 7 cas (17,5%)

**Tableau 3:** Type de fractures après la chute

Types de fractures	Nombre de cas /40 (%)	Genre	Age (ans)	DMO
Fractures	13 (32,5%)	12F/1H	75 (moyenne)	
Poignet	4	4 F	71-81-70-85	NF-OP-OP-OP
Col du fémur	3	3 F	82-77-84	NF-OP-OP
Avant-bras	2	F-H	85-73	OP-NF
Col de l’humérus	1	F	69	OP
Humérus	1	F	80	OP
Métatarsienne	1	F	72	NF
Avant pied	1	F	65	OP
Tassement vertébral	1	F	65	OP

**NF**: non fait, **OP**: ostéoporose

### Conséquences psychosociales des chutes

Les conséquences psychosociales ont été observées chez tous nos patients et sont détaillées dans le **Tableau 4**. Elles étaient plus fréquentes chez les femmes. Un séjour prolongé au sol supérieur à une heure a été rapporté par un seul patient parmi 5 qui ont eu un syndrome post chute.

**Tableau 4:** Conséquences psychosociales des chutes

Type de conséquences	Nombre de cas 40 (%)	Genre
Peur de rechuter	23 (57,5%)	16F/ 7H
Sentiment de tristesse après la chute	19 (47,5%)	12F/7H
Limitation de l’activité	18 (45%)	12F/6H
Peur de remarcher	18 (45%)	11F/7H
Syndrome post chute	5 (12,5%)	3F/2H

### Étude de l'autonomie selon le score ADL après la chute

Pour tous les patients, le score ADL a été calculé après la chute au moment de notre entrevue avec eux. La valeur moyenne du score ADL était de 4,8 (±1,46) avec des extrêmes allant de 0 à 6. La répartition du score ADL en fonction du nombre des patients est détaillée dans le **Tableau 5**. Une perte de l'autonomie, identifiée à partir d'un score ADL ≤5, a été objectivée chez 16 patients (40%). Parmi ces 16 patients, un séjour prolongé au sol a été notée chez 2 patients (un homme et une femme). L'évaluation de l'aide pour les activités de la vie quotidienne après la chute a montré que 16 patients (40%) avaient besoin d'une tierce personne (enfants, mari, belle fille). Lors de nos entretiens avec les malades, nous avons constaté que 17 patients consultaient seuls et le reste des malades soit 23 étaient en compagnie d'une tierce personne.

**Tableau 5:** Répartition des patients en fonction du score ADL

Score ADL	Nombre de patients / 40
**0**	1
**2**	2
**2,5**	2
**3**	2
**4**	1
**4,5**	3
**5**	5
**5,5**	9
**6**	15

### Étude de la marche après la chute

Une reprise de la marche sans aide après la chute a été constatée chez 24 patients (60%). Seize malades avaient nécessité une aide pour remarcher, par canne dans 9 cas, béquilles dans 4 cas, chaises roulantes dans 2 cas et une tierce personne dans 1 cas. Après la chute, 17 patients (42,5%) ont limité leurs activités au domicile. Le reste des patients soit 23 (57,5%) ont gardé une activité sociale avec des sorties dans le but de visites familiales (23 cas) et pour faire des courses (17 cas). Vingt-trois patients se déplaçaient à pieds et en voiture. Neuf patients étaient capables d'utiliser les transports en commun.

## Discussion

Les conséquences des chutes chez nos patients ont été dramatiques sur le plan physique et psychique avec perte de l'autonomie dans 40% des cas. Nous avons trouvé une nette prédominance des fractures en post chutes chez les femmes (12F/1H) probablement expliquée par la nette prédominance féminine dans notre étude mais également par l'ostéoporose chez nos patientes qui ont été explorées par la DMO. L'existence d'autres facteurs de risque surajoutés au sexe féminin et à l'ostéoporose constitue les facteurs avancés dans la littérature pour expliquer la fréquence plus importante des fractures chez les femmes [9-12].

Dans notre série, les fractures des membres supérieurs ont été les plus fréquentes probablement à cause du faible effectif. Les fractures de hanches n'ont pas été rapportées chez nos patients contrairement à la littérature ou les fractures de hanches sont les plus fréquentes puis en deuxième position les fractures des membres puis les traumatismes crâniens et les fractures vertébrales [9-16]. Des conséquences non graves ont été rapportées par 26 de nos patients (65%). En se basant sur les populations chuteuses étudiées, 22 à 60% ont une blessure après la chute et 10 à 15% ont des blessures graves [10]. Les blessures les plus fréquemment rapportées sont les abrasions, les contusions et les entorses [10, 17-19].

Les conséquences psychosociales sont aussi importantes, mais plus difficiles à quantifier. Après la chute, 57,5% de nos patients ont déclaré une peur de rechuter et 47,5% un sentiment de tristesse après la chute. Notre résultat est en concordance avec les données de la littérature où 19 à 85% des patients ayant fait une chute dans l'année ont déclaré redouter une récidive [4, 20-24]. Dans notre étude, 45% ont avoué une limitation de l'activité après la chute. Notre résultat dépasse légèrement les résultats rapportés dans la littérature où la peur de rechuter entraine une réduction de l'activité dans 26% à 35% des cas [4, 25]. Dans notre série comme dans la littérature, la femme parait plus concernée que l'homme par cette complication, ceci est probablement dû aux complications graves plus fréquentes chez elles [26]. Le syndrome post chute est une des complications redoutables à cause de la perte d'autonomie importante qu'il peut engendrer. Il a été observé dans 12,5% des cas dans notre étude. Il n'y a pas de données épidémiologiques concernant ce paramètre dans la littérature.

Au final, malgré son caractère rétrospectif, notre étude reste intéressante car d'une part elle s'est intéressée aux conséquences traumatiques et psychosociales des chutes et d'autre part elle a témoigné de façon indirecte sur le retentissement sur la qualité de vie chez le sujet âgé et le cout qu'elle pourrait avoir en Tunisie.

## Conclusion

L'aménagement du domicile, le port de vêtements et de chaussures adaptés ainsi qu'une prise en charge adéquate de toutes les pathologies pouvant entrainer un trouble aigu de l'équilibre constituent des moyens de prévention des chutes et de leur conséquence. L'apprentissage du relevé du sol en post chutes constitue un moyen simple de prévention visant à améliorer la qualité de vie du sujet âgé et diminuer le cout élevé de la prise en charge secondaire aux conséquences de chutes.

### Etat des connaissances actuelles sur le sujet

- Les chutes chez le sujet âgé constituent un problème de santé mondial de par les complications traumatiques et psychosociales qui peuvent entrainer une perte de l'autonomie et un état de dépendance;
- Les conséquences traumatiques des chutes sont plus fréquentes chez les femmes et dominées par les fractures de hanche;
- Les conséquences psychosociales des chutes sont plus difficiles à quantifier. Parmi eux, le syndrome post chute constitue la complication la plus redoutable.

### Contribution de notre étude à la connaissance

- Les conséquences traumatiques des chutes ont été plus fréquentes chez les femmes et étaient dominées par les fractures au niveau des membres supérieurs;
- Les conséquences psychosociales ont été observées chez tous les chuteurs. Par conséquent, elles doivent être systématiquement recherchées pour éviter la progression vers un syndrome post chute;
- Un bilan détaillé des circonstances des chutes et du temps de séjour au sol en post chutes permettra d'adapter une prise en charge adaptée à chaque patient.

## Conflits d’intérêts

Les auteurs ne declarent aucun conflits d'intérêts.
